# Social Media Use Motives and Mental Health Among Adolescents: Cross-Sectional Study

**DOI:** 10.2196/111020

**Published:** 2026-10-28

**Authors:** James Lian, Morgane Künzi, Jack L Andrews, John Gallacher, Sarah Bauermeister

**Affiliations:** 1 Department of Psychiatry, University of Oxford, Warneford Lane, Oxford, England, OX3 7JX, United Kingdom, 61 449583338; 2 Department of Experimental Psychology, University of Oxford, Oxford, England, United Kingdom

**Keywords:** digital mental health, motivation, school mental health, structural equation modeling, SEM, problematic social media use, adolescent mental health, social media

## Abstract

**Background:**

Social media use is ubiquitous among young people, yet its relationship with mental health may depend on why it is used. Understanding the motives underlying social media engagement may provide greater insights into its potential risks and benefits than considering overall use alone.

**Objective:**

This study aimed to identify latent dimensions of motives for social media use and examine their associations with anxiety, depression, well-being, and resilience in a sample of older adolescents.

**Methods:**

Cross-sectional data were drawn from the BrainWaves study (N=6820; mean age 16.72, SD 0.74 years). Social media use motives were assessed using 10 items and exploratory factor analysis identified 2 latent dimensions: avoidant-escapist use (boredom, addiction, distraction, and procrastination) and social-recreational use (socializing, communication, entertainment, and observation). Confirmatory factor analysis was used to estimate factor scores, which were examined using linear regression. Structural equation modeling estimated associations with anxiety, depression, well-being, and resilience, with secondary analyses accounting for gender and deprivation.

**Results:**

Linear regression analyses showed that avoidant-escapist motives were associated with higher anxiety and depressive symptoms, whereas social-recreational motives were associated with lower symptoms. No interactions with gender or deprivation were observed. The structural equation model showed acceptable model fit (comparative fit index=0.945; root mean squared error of approximation=0.057; standardized root mean squared residual=0.054). Avoidant-escapist motives were associated with higher anxiety (β=0.543) and depressive symptoms (β=0.629) and lower well-being (β=−0.610) and resilience (β=−0.469). Conversely, social-recreational motives were associated with lower anxiety (β=−0.228) and depressive symptoms (β=−0.342) and higher well-being (β=0.362) and resilience (β=0.242; *P*<.001 in all cases). These patterns remained consistent after accounting for gender and deprivation.

**Conclusions:**

Social media use shows different associations with older adolescent mental health depending on underlying motives. Focusing on why young people engage with social media rather than on overall use alone may provide a more informative framework for understanding potential risks and benefits. Given the cross-sectional design, causal inferences cannot be made.

## Introduction

Social media use among young people has been increasing rapidly in recent years [1], and this has mirrored a trend of increasing mental health problems among this age group [2]. Most adolescents today own devices with internet access, which enable them to be online from anywhere and at any time [3]. Many of them communicate online through social media accounts (eg, Instagram, Snapchat, and TikTok), which are platforms that allow individuals to connect with others within a virtual network. For some adolescents, social media use may confer benefits such as facilitating social interactions, access to support networks and communities, and creative expression and entertainment [4]. However, for others, social media use may confer risks such as exposure to cyberbullying and negative social comparisons [3]. Given this, there is considerable public concern surrounding how social media use might affect youth mental health and the extent to which it explains rising rates of mental health problems among young people.

Much of this concern comes from increasing evidence pointing to associations between social media use and negative mental health outcomes among young people. For example, a recent meta-analysis of 140 studies found that digital media use was consistently associated with higher depression, substance use, internalizing and externalizing disorders, and self-injurious thoughts in young populations [5]. Similarly, another meta-analysis found moderate associations between problematic social media use and depression, anxiety, and stress in adolescents [6]. In addition, a study found associations between time spent on social media and poorer sleep, lower self-esteem, and body image dissatisfaction, factors linked to poorer mental health [7]. Therefore, evidence suggests that social media use has the potential to directly and indirectly impact mental health outcomes through multiple pathways.

While these findings are cause for concern, they rely on crude measures of social media use (eg, time spent), which obscures a more nuanced perspective. Indeed, findings from the literature examining the relationship between *how* adolescents use social media and mental health are mixed. For example, a meta-analysis of 141 studies found that both active (posting and interacting) and passive (browsing and scrolling) social media use were not, on average, related to mental health and well-being outcomes [8]. Importantly, the authors observed that patterns varied widely by context, such as user age, demographic differences, type of social media used, and how it was used. Recently, attention has turned toward understanding the underlying motives that drive adolescent engagement with social media [9], that is, to ask why adolescents are drawn to use social media in the first place. However, relatively little research has examined the relationship between social media use motives and mental health outcomes in younger populations.

Therefore, understanding the motives behind social media use is emerging as a key factor in understanding its relationship with mental health. For example, one study found that intrapersonal motives such as distracting oneself and passing time were more strongly linked to problematic social media use relative to other motives such as relationship maintenance [10]. Findings have also indicated that using social media for entertainment purposes predicted greater depression and generalized anxiety, while motivation for following and monitoring others was associated with higher social anxiety [11]. Another study found that motivation to use social media to build and maintain relationships with others predicted satisfaction with friends and family as well as lower internalizing psychopathological symptoms [12]. In addition, social media can also be helpful as a coping tool through distracting oneself from stressful thoughts, venting emotions, and aiding in solving the stress-evoking problem. However, this relationship is complex as using social media for escapism may also be linked to poorer mental health given that it impedes the adoption of more adaptive solution-focused coping strategies [13]. In addition, the use of escapism as a coping strategy for daily stressors may also increase the risk of problematic social media use [14,15].

Evidently, research examining the motives behind social media use and adolescent mental health outcomes is complex and inconsistent, possibly due to several key limitations with the way in which motives are measured. For example, motives are often poorly defined and lack data-driven justification, typically operationalized as individual items or summed scores that assume equal weighting across behaviors [16]. Such approaches fail to capture the underlying latent structure of social media use and may obscure meaningful distinctions between different patterns of engagement. In addition, studies rarely account for important contextual factors such as gender, resilience, and deprivation and are often based on relatively small or unrepresentative samples of school-aged populations [6,17]. These limitations hinder the ability to disentangle the complex and potentially opposing associations between different forms of social media use and mental health outcomes.

Structural equation modeling (SEM) provides a useful framework to address limitations in operationalizing digital use and accounting for covariates. SEM enables the identification of latent dimensions of social media use, accounting for measurement error, and the estimation of multiple relationships simultaneously within a unified model [18]. This approach allows for a more nuanced understanding of how distinct patterns of social media use relate to mental health.

As such, the goal of this study was to examine the associations between different motives of social media use and mental health outcomes (anxiety, depression, well-being, and resilience) in a sample of older adolescents. We hypothesized that the social media use motive items would reflect latent dimensions broadly representing adaptive and maladaptive patterns of use. We further expected that maladaptive social media use motives would be associated with higher anxiety and depressive symptoms and lower well-being and resilience, whereas adaptive motives would be associated with lower anxiety and depressive symptoms and higher well-being and resilience.

## Methods

### Participants

The data come from the BrainWaves study [19]. BrainWaves is a longitudinal cohort study that collects data on UK students aged 16 years and older. Three waves have been collected to date (wave 1: April 2024; wave 2: November 2024; wave 3: November 2025), with data collection ongoing. BrainWaves includes data on demographics and a broad collection of variables, including mental health, well-being, lifestyle, cognition, physical health, adversity, and relationships.

The sample used for this study was drawn from wave 3, when social media use motives were assessed, and included 11,498 participants. Participants with complete data on all social media use motive items were retained for the primary analyses. Analyses incorporating gender and deprivation were restricted to participants with available data on these covariates.

### Ethical Considerations

Ethics approval for the BrainWaves study was granted by the Central University Research Ethics Committee (R91383/RE001; amendment R91383/RE004). The study was conducted in accordance with the Declaration of Helsinki, and all participants provided written informed consent prior to participation. Participant data were stored and accessed within the BrainWaves Informatics Hub, which operates within the Dementias Platform UK Data Portal Trusted Research Environment, with controlled access procedures in place to protect participant privacy and confidentiality. Participants did not receive compensation for participation.

### Outcome Measures

#### Motivations to Use Social Media

Motivations to use social media were assessed using the following question: “Why do you think you spend time on social media?” with 10 listed motives and binary responses (0=no; 1=yes). The listed motives were “nothing else to do, boredom;” “addictive;” “fun, leisure, entertainment;” “as a distraction from problems;” “procrastination from tasks;” “to make money/for payment;” “to socialize with others;” “to communicate with friends and family;” “seeking help or advice online;” and “to see what others are doing.” The items “other” and “not sure” were not included in our analyses.

#### Anxiety and Depression

Anxiety and depression were measured using the 11-item version of the Revised Child Anxiety and Depression Scale [20]. The 11-item version comprises 6 anxiety symptom items and 5 depressive symptom items. The McDonald ω coefficients in the community sample range from 0.87 to 0.94. Anxiety and depression symptom scores were calculated by summing the respective items. The anxiety symptom score ranges from 0 to 18, and the depressive symptom score ranges from 0 to 15, with higher scores representing greater symptom severity.

#### Well-Being

Well-being was measured using the Short Warwick-Edinburgh Mental Wellbeing Scale (SWEMWBS) [21]. The SWEMWBS demonstrated internal consistency in an adolescent Norwegian population, with a Cronbach α value of 0.88 [22]. The 7 items of the scale were added together and converted to a metric score using the Rasch model [22]. The score ranges from 7 to 35, with higher scores representing higher well-being.

#### Resilience

Resilience was measured using the Brief Resilience Scale (BRS) [23]. Cronbach α coefficients for the BRS range from 0.80 to 0.91. Items 2, 4, and 6 were reverse coded, and all 6 items were summed and divided by 6. The score ranges from 1 to 5, with a higher score representing greater resilience.

#### Covariates

Gender was assessed using 1 item: “How do you describe your gender?” with 8 response options (“female,” “male,” “non-binary or gender diverse,” “other gender,” “trans,” “in another way,” “unsure,” or “prefer not to answer”). The item was dichotomized (0=female; 1=male). Participants selecting other categories were excluded from analyses due to small numbers and the heterogeneity of these groups, which limited statistical power and precluded meaningful interpretation as a single combined category.

To measure social and economic deprivation, 3 items were used: “As far as you are aware, has your family used a food bank in the last year?” “As far as you are aware, have you ever been at risk of homelessness?” and “Are you currently entitled to free school meals?” On the basis of these items, a deprivation variable was created, with scores ranging from 0 to 3.

### Statistical Analyses

Exploratory factor analysis (EFA) was used to identify latent dimensions of social media use motives. We applied Geomin rotation with tetrachoric correlations and weighted least squares mean and variance adjusted (WLSMV) estimator to account for binary indicators. Model selection was guided by model fit indexes, parsimony, and factor interpretability [24]. Specifically, factors should be composed of at least 3 items, with loading thresholds of 0.40 or higher [25,26]. Items with substantial cross-loadings or low primary loadings were removed. The full sample size was used for the EFA, but the results (which remain unchanged) from a half-random split of the sample are also reported in Multimedia Appendix 1.

Confirmatory factor analysis (CFA) was used to estimate individual-level factor scores based on the selected factors from EFA. Again, the full sample size was used, and results from the other random split are reported in Multimedia Appendix 1. The models were estimated using the WLSMV estimator to accommodate the binary indicators [27]. Model fit was assessed using the comparative fit index (CFI; ≥0.95), root mean squared error of approximation (RMSEA; <0.06), and standardized root mean squared residual (SRMR; <0.08) [28].

To aid interpretability and visualize associations, simple correlations were visualized using linear plots between social media use motive factor scores and anxiety and depressive symptoms. Linear regression models were subsequently used to estimate the strength of these associations while adjusting for gender and deprivation. Interaction terms were included to examine whether associations between social media use motives and mental health outcomes varied by gender or deprivation.

SEM was used to simultaneously estimate the relationships between latent social media use constructs and multiple dimensions of mental health outcomes within a unified framework. In this model, the latent variables of social media use motives were predictors of anxiety, depression, well-being, and resilience. All error terms of the outcomes were allowed to covary, and the latent factors were also allowed to correlate. In addition to the fitted model, a second model including covariates (as predictors of social media use motives and anxiety, depression, well-being, and resilience) was also fitted and reported in Multimedia Appendix 1. Results from the split sample can be found in Multimedia Appendix 1.

The models were estimated using the WLSMV estimator to accommodate the mix of binary and continuous indicators [27]. The same model fit indexes as in the CFA were used (ie, CFI, RMSEA, and SRMR). Missing data on endogenous variables were accommodated using available data under WLSMV estimation. As Mplus treats observed exogenous covariates as fixed variables, participants with missing gender or deprivation data were excluded from models containing these covariates.

## Results

### Descriptive Statistics

Of the 11,498 wave 3 participants, 6820 had complete data on all social media use motive items and were included in the primary analyses. Participants ranged from 16 to 20 (mean 16.72, SD 0.74) years, with only 4 participants aged 20 years; the sample therefore predominantly represented older adolescents. Analyses incorporating gender and deprivation included 5540 participants with available covariate data. Among participants with available deprivation data, 88.3% (5114/5795) reported none of the 3 deprivation indicators, signaling a highly skewed distribution (Table 1).

**Table 1.** Descriptive statistics of the variables included in the model (N=6820).

Variables	Values
Age (y), mean (SD; range)	16.72 (0.74; 16-20)
Gender (male), n (%)	36.9
Ethnicity (non-White), n (%)	21
Well-being (7-35), mean (SD; range)	21.14 (3.37; 7-35)
Resilience (1-5), mean (SD; range)	3.02 (0.78; 1-5)
Deprivation (0-3), mean (SD; range)	0.15 (0.44; 0-3)
0, n (%)	75
1, n (%)	8
2, n (%)	1.6
3, n (%)	0.5
Missing, n (%)	15
Social media questionnaire (true), n (%)	
Boredom	62
Addiction	36
Entertainment	73
Distraction	35.5
Procrastination	57.4
Socialization	58.5
Communication	70.3
Observation	32.7

### EFA

Initially, a 3-factor solution demonstrated the best fit but was not retained because the third factor was defined by only 2 items (“making money” and “seeking help”), limiting its interpretability and robustness (Table S1 in Multimedia Appendix 1). Accordingly, a 2-factor solution was selected as the most parsimonious and conceptually meaningful representation of the data (Table 2). The 2-factor solution distinguished between avoidant-escapist and social-recreational motives for social media use.

**Table 2.** Geomin rotated loading for exploratory factor analysis (2 factors; N=6820).

Indicator	Factor 1	Factor 2
Boredom	*0.388* ^a^ ^,^ ^b^	0.062^b^
Addiction	*0.668* ^b^	–0.035
Entertainment	0.073^b^	*0.506* ^b^
Distraction	*0.692* ^b^	–0.006^b^
Procrastination	*0.720* ^b^	0.118^b^
Money	0.031	0.180^b^
Socialization	0.002	*0.781* ^b^
Communication	–0.019	*0.847* ^b^
Seeking help	0.313^b^	0.321^b^
Observing others	0.263^b^	*0.498* ^b^

a Italics indicate an item retained for the factor.

b Significant loading on factor.

The boredom item loaded primarily on the avoidant-escapist factor (loading=0.388) but fell marginally below our prespecified loading threshold of 0.40. We retained the item because it was conceptually consistent with the avoidant-escapist construct and showed minimal cross-loading on the social-recreational factor (loading=0.062). As this represented a deviation from our prespecified criterion, sensitivity analyses were conducted excluding boredom; the 2-factor structure and CFA model fit remained substantively unchanged (CFI=0.978; RMSEA=0.046; SRMR=0.039; Table S5 and Figure S4 in Multimedia Appendix 1).

The final avoidant-escapist factor comprised 4 items reflecting disengagement and avoidance processes (“boredom,” “addictive use,” “distraction from problems,” and “procrastination”), while the social-recreational factor comprised 4 items reflecting social engagement and recreational use (“entertainment,” “socialising,” “communication with friends and family,” and “observing others”). All retained items loaded onto their respective factors (Table 2).

### CFA

Results from CFA based on the 2 selected factors from EFA (ie, avoidant-escapist and social-recreational) also showed good fit (N=6820; CFI=0.977; RMSEA=0.041; SRMR=0.038). The correlation between the 2 latent factors was *r*=0.519 (*P*<.001; Figure S1 in Multimedia Appendix 1). Factor scores derived from CFA were used in subsequent visualization and regression analyses.

### Linear Regression

Figure 1 illustrates the unadjusted associations between social media use motive factor scores and mental health outcomes. Avoidant-escapist social media use showed clear positive associations with both depressive and anxiety symptoms. In contrast, associations between social-recreational use and mental health outcomes were positive but comparatively weaker in the unadjusted plots.

**Figure 1.**
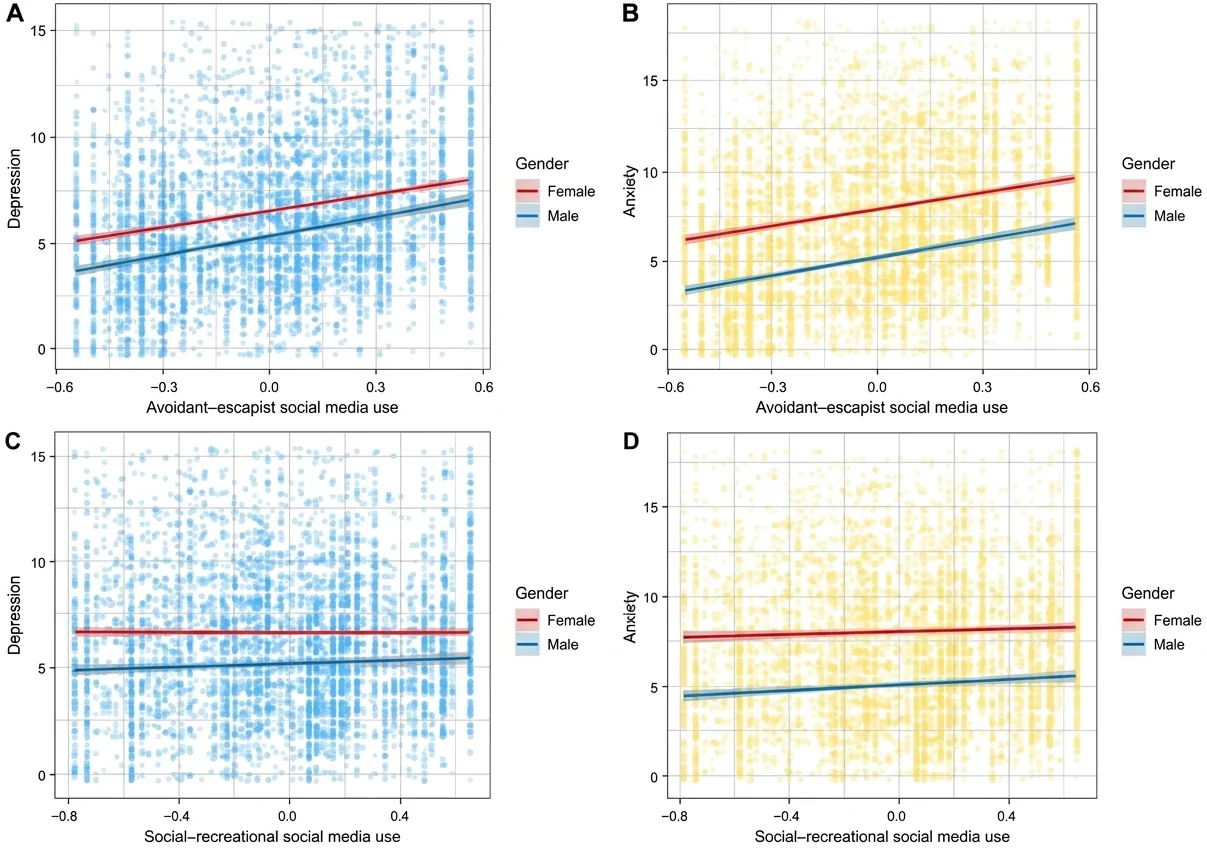
Associations between social media use motive factor scores and depression and anxiety outcomes. (A) Avoidant-escapist use and depression. (B) Avoidant-escapist use and anxiety. (C) Social-recreational use and depression. (D) Social-recreational use and anxiety.

Linear regression analyses adjusting for gender, deprivation, and shared variance between dimensions demonstrated that avoidant-escapist social media use was significantly associated with greater anxiety and depressive symptoms, whereas social-recreational use was associated with lower levels of both outcomes (Figure 1 and Table 3). Associations were stronger for avoidant-escapist compared to social-recreational use. Being male was associated with lower anxiety and depressive symptoms relative to being female, and higher levels of deprivation were also associated with higher anxiety and depressive symptoms. No evidence of interaction between gender or deprivation and social media use motives was observed (*P*>.05 in all cases).

**Table 3.** Associations between social media use motives and mental health outcomes from linear regression models, including interactions with gender and deprivation.

Predictor	Anxiety, *B*^a^ (95% CI)	*P* value	Depression, *B* (95% CI)	*P* value
Avoidant-escapist	4.68 (4.28 to 5.07)	<.001	4.46 (4.14 to 4.78)	<.001
Social-recreational	–1.78 (–2.09 to –1.48)	<.001	−2.08 (–2.33 to –1.83)	<.001
Gender (male)	–2.74 (–2.94 to –2.55)	<.001	−1.19 (–1.35 to –1.03)	<.001
Deprivation	0.91 (0.68 to 1.13)	<.001	0.98 (0.80 to 1.16)	<.001
Avoidant-escapist × gender	–0.21 (–1.04 to 0.61)	.61	0.12 (–0.56 to 0.79)	.73
Social-recreational × gender	0.58 (–0.04 to 1.21)	.07	0.48 (–0.03 to 0.99)	.06
Avoidant-escapist × deprivation	–0.29 (–1.33 to 0.74)	.58	0.07 (–0.72 to 0.87)	.86
Social-recreational × deprivation	–0.10 (–0.90 to 0.69)	.80	–0.05 (–0.66 to 0.57)	.88

a Unstandardized coefficient.

### Structural Equation Model

#### Model 1 Without Covariates

The model demonstrated acceptable fit (N=6820; CFI=0.945; RMSEA=0.057; SRMR=0.054). Avoidant-escapist social media use was associated with poorer mental health outcomes, including higher anxiety (β=0.543) and depressive (β=0.629) symptoms, as well as lower well-being (β=−0.610) and resilience (β=−0.469; *P*<.001 in all cases). In contrast, social-recreational use was associated with more favorable outcomes, including lower anxiety (β=−0.228) and depressive (β=−0.342) symptoms and higher well-being (β=0.362) and resilience (β=0.242; *P*<.001 in all cases; Figure 2).

**Figure 2.**
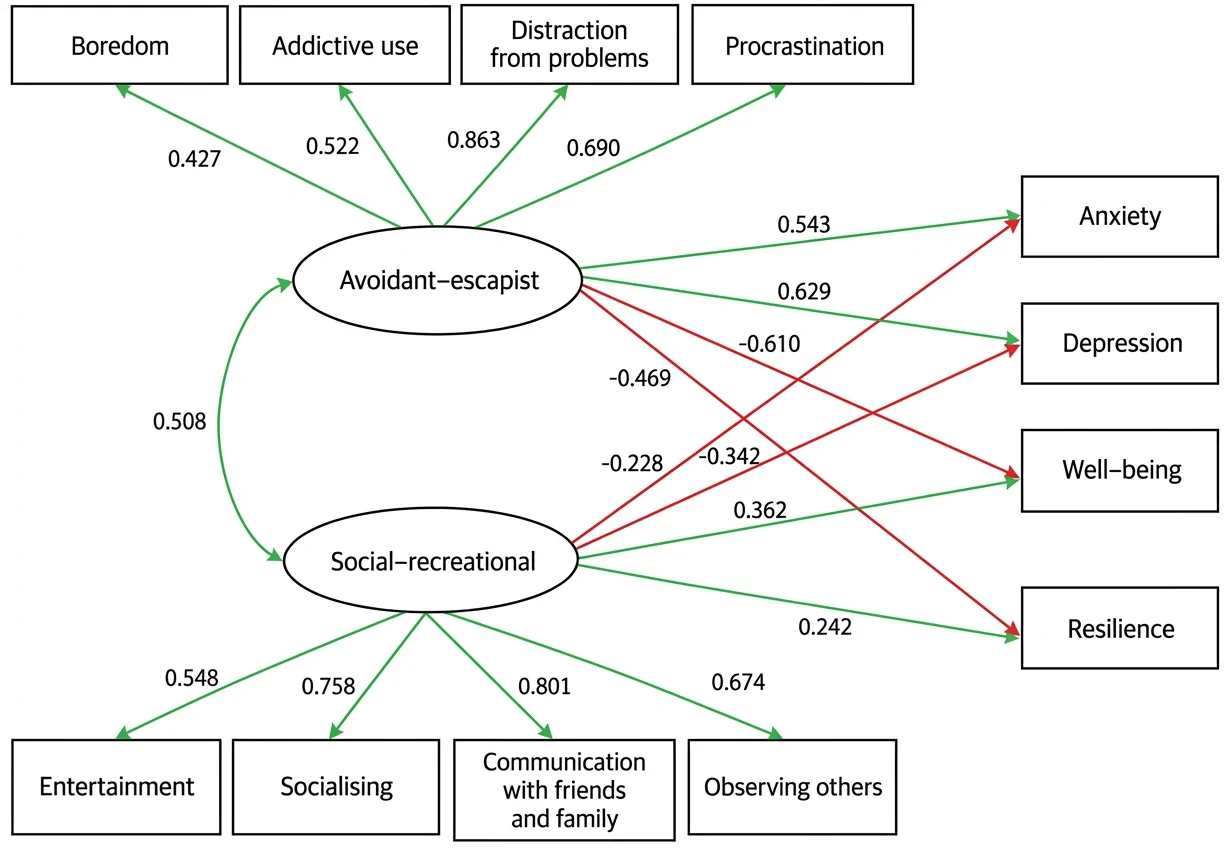
Illustration of the fitted structural equation model. For clarity purposes, the covariances between variables were not drawn. All the paths drawn were significant, with *P*<.001.

Avoidant-escapist and social-recreational social media use were moderately correlated (*r*=0.508; *P*<.001), indicating that these patterns of use co-occur. Furthermore, the error terms of anxiety and depression were positively correlated (*r*=0.598; *P*<.001), while both were negatively associated with well-being (anxiety: *r*=–0.444 and *P*<.001; depression: *r*=–0.533 and *P*<.001) and resilience (anxiety: *r*=–0.488 and *P*<.001; depression: *r*=–0.390 and *P*<.001).

#### Model 2 With Covariates

For the model including gender and deprivation as predictors of social media use motives and anxiety, depression, well-being, and resilience, the model demonstrated good fit (n=5540; CFI=0.950; RMSEA=0.051; SRMR=0.048). Gender was significantly associated with both social media use motives and mental health. Female individuals reported higher levels of both avoidant-escapist (β=−0.228) and social-recreational (β=−0.148) use, as well as higher anxiety (β=−0.292) and depressive (β=−0.134) symptoms, whereas male individuals reported higher well-being (β=0.157) and resilience (β=0.254; *P*<.001 in all cases). Deprivation was significantly associated with both social media use motives and mental health. Higher deprivation scores were associated with higher levels of avoidant-escapist use (β=0.042; *P*=.02) and higher levels of anxiety (β=0.074; *P*<.001) and depressive (β=0.103; *P*<.001) symptoms. Lower deprivation scores were associated with higher levels of social-recreational use (β=−0.046; *P*=.007) and higher well-being (β=–0.079; *P*<.001) and resilience (β=–0.049; *P*<.001; Table S2 and Figure S2 in Multimedia Appendix 1).

## Discussion

### Principal Findings

Our study used data from a large-scale cohort of older adolescents in the United Kingdom to examine associations between social media use and mental health. By applying EFA, CFA, and SEM, we identified underlying latent dimensions of social media use motives, accounting for measurement error, and estimated multiple relationships simultaneously within a unified framework. This approach goes beyond traditional methods that rely on individual items or summed scores, allowing for a more nuanced understanding of how distinct patterns of social media engagement relate to mental health outcomes.

On the basis of our EFA, we identified 2 underlying dimensions of social media use across 8 items: avoidant-escapist and social-recreational motives. These dimensions broadly align with findings from prior attempts to classify motivations of social media use [29,30]. For example, previous reviews have identified categories of motives related to relationship maintenance, entertainment, information seeking, and emotional regulation [31]. However, our findings suggest that motives that are grouped together under broader categories (eg, entertainment and escapism considered as positive sensation seeking) [9] may instead reflect psychologically distinct patterns of engagement with different associations with mental health. As such, applying a data-driven approach to categorizing social media use motives may provide a more informative framework to understanding its relationship with adolescent well-being.

Across both linear and structural equation models, we found that avoidant-escapist motives were associated with poorer mental health (ie, higher anxiety and depressive symptoms and lower well-being and resilience). Similar patterns were found in a study where escapist motivations for social media use were linked with higher self-reported stress [32]. Similarly, nonsocial motivations of social media use were associated with internalizing psychopathological symptoms in a Spanish population [12]. Furthermore, the literature has shown that digital use driven by procrastination and addiction is associated with poorer mental health [33]. These trends are likely explained by unfavorable social comparisons [34], worsening symptoms for those with existing mental health conditions [35], and disruptions in daily activities or social life due to excessive or uncontrolled engagement with social media [36]. Social media platforms are inherently rewarding environments that provide entertainment, distraction, and social reinforcement, which may help explain why adolescents continue engaging with them despite potential negative consequences [37].

In contrast, social-recreational motives were associated with better mental health outcomes (ie, lower anxiety and depressive symptoms and higher well-being and resilience). These results are consistent with literature reporting positive benefits of social media use in social contexts [38]. Often, social media is used to share personal experiences, keep up with friends, and give or receive support to others [39]. As such, it appears that social connections developed and fostered through social media play an important role in promoting young people’s well-being [40]. For example, time spent playing video games has been shown to be one of the most effective ways for adolescents to generate positive feelings due to its social and emotional benefits [41]. However, it is important to consider that such motives are not inherently positive; for example, entertainment could be linked to problematic smartphone use [42]. Specifically, social media provides positive reinforcement for continued use, which increases engagement and may lead to less controlled use and addiction.

In terms of gender differences, we found that female individuals reported more of both social-recreational and avoidant-escapist social media use and also exhibited higher anxiety and depressive symptoms relative to male individuals. In contrast, male individuals reported higher overall well-being and resilience relative to female individuals. These results may be explained by gender differences in the coping strategies used that influence digital use and lead to different outcomes in problematic social media use [43]. Our results are consistent with those of previous studies that report gender differences in social media use [44,45]. For example, prior work has shown that girls primarily use social media to reinforce preexisting friendships, while boys mainly use these sites to flirt and make new friends [4,15]. Interestingly, we found no significant interactions between gender and social media use motives on mental health. This suggests that the associations between social media use motives and mental health outcomes are broadly consistent across genders despite differences in overall levels of use and symptom severity.

We found that higher levels of deprivation were associated with higher levels of avoidant-escapist use and greater anxiety and depressive symptoms. These results are consistent with those of a study that found greater negative impacts of social media use on adolescents with low socioeconomic status compared to adolescents with high socioeconomic status [46]. We also observed that low levels of deprivation were associated with more social-recreational motives and higher well-being and resilience, consistent with the findings of Kurten et al [47]. However, Kurten et al [47] also found that materially deprived adolescents (ie, those who lack resources for material participation in mainstream society) tend to spend more time on social media overall compared to those less deprived. Our findings therefore suggest that preexisting vulnerabilities such as deprivation may be related to motivations for social media use and their associations with mental health. However, we note that these covariate-related findings should be interpreted in light of the reduced analytic sample as participants excluded due to missing covariate data had poorer mental health on average than those retained (Table S7 in Multimedia Appendix 1).

### Limitations

Several limitations should be considered when interpreting these findings. First, the cross-sectional design precludes causal inference, and the observed associations may be bidirectional. For example, adolescents with preexisting psychological vulnerabilities may be more likely to engage with social media for avoidant or escapist reasons [35], while repeated engagement in these patterns could reinforce avoidance-based coping over time [48]. Longitudinal and experimental research is therefore needed to establish temporal relationships between social media use motives and mental health.

Second, the sample predominantly comprised older adolescents aged 16 to 19 years, with only 4 participants aged 20 years and no participants younger than 16 years. The findings should therefore not be assumed to generalize to younger adolescents, for whom patterns and functions of social media use may differ.

Third, inclusion of gender and deprivation in the covariate-adjusted structural equation model reduced the analytic sample from 6820 to 5540 because participants with missing predictor data were excluded. Participants excluded from this model reported higher anxiety and depressive symptoms and lower well-being and resilience on average than those retained. This pattern indicates potential selection bias, and estimates from the covariate-adjusted model may therefore underrepresent individuals experiencing poorer mental health. In addition, deprivation was highly zero-inflated, with 88.3% (5114/5795) of participants with available data reporting none of the 3 deprivation indicators. As such, associations involving deprivation should be interpreted cautiously.

Finally, social media use was assessed using self-report and may therefore be subject to reporting bias. Previous research suggests that discrepancies between self-reported and objectively measured social media use may vary according to social media platform and respondent gender [49].

### Future Studies

Longitudinal and experimental studies are needed to investigate temporal directionality and causal mechanisms. Furthermore, it is important to examine underrepresented groups (nonbinary and transgender individuals and ethnic minority groups) to better understand and serve these populations. Understanding these associations and their dynamics is necessary to make the most of the benefits of social media while preventing risks and harms.

### Conclusions

The results of our study suggest that social media use motives among older adolescents can be characterized by 2 broad dimensions (avoidant-escapist and social-recreational), which are differently associated with mental health. Our findings emphasize the importance of considering how and why young people use digital tools to better understand their relationship with mental health. Notably, social media use in youth is not uniformly harmful or beneficial, and its associations with mental health depend on underlying motives. Further research in this area is critical, especially as many institutions and governments worldwide are considering outright social media bans in schools and for young people [50]. Although often linked with adverse outcomes, social media use is nuanced and often offers benefits to its users. As such, total bans may not achieve the desired goals and may even lead to iatrogenic outcomes [51]. Interventions and guidelines should therefore move beyond screen time and consider patterns of engagement when assessing risk or benefit. Longitudinal and experimental research, particularly in diverse and underrepresented groups, is needed to better understand associations between digital use and mental health in younger populations.

## Supplementary material

10.2196/111020 Multimedia Appendix 1 Supplementary tables and figures.

## Abbreviations

BRS: Brief Resilience Scale
CFA: confirmatory factor analysis
CFI: comparative fit index
EFA: exploratory factor analysis
RMSEA: root mean squared error of approximation
SEM: structural equation modeling
SRMR: standardized root mean squared residual
SWEMWBS: Short Warwick-Edinburgh Mental Wellbeing Scale
WLSMV: weighted least squares mean and variance adjusted
