# Supplementary material for: Social Media Use Motives and Mental Health Among Adolescents: Cross-Sectional Study
Source: JMIR Ment Health. 2026 Oct 28;13:e111020. doi: 10.2196/111020 (PMC13717429; doi:10.2196/111020)
Supplement: Supplementary file 1 [file mental-v13-e111020-s001.docx]

**Table S1**

*Geomin rotated loading for Exploratory Factor Analysis (three-factor) (N = 6820).*

| Indicator | | Factor 1 | Factor 2 | Factor 3 |
| --- | --- | --- | --- | --- |
| 1 | Boredom | 0.113* | **0.362*** | 0.016 |
| 2 | Addictive | -0.015 | **0.489*** | 0.269* |
| 3 | Entertainment | **0.519*** | 0.012 | 0.028 |
| 4 | Distraction | 0.008 | **0.493*** | 0.303* |
| 5 | Procrastination | 0.208* | **0.739*** | -0.003 |
| 6 | Money | 0.002 | -0.415* | **0.698*** |
| 7 | Socialise | **0.785*** | -0.061 | 0.005 |
| 8 | Communication | **0.936*** | -.003 | -0.177* |
| 9 | Seeking help | 0.250* | -.000 | **0.473*** |
| 10 | Observing others | **0.489*** | 0.074 | 0.231* |

*Note*: Asterisk indicates significant loading on factor; bold text indicates an item retained for the factor.

**Table S2**

*Geomin rotated loading for Exploratory Factor Analysis (three-factor) (N = 3410).*

| Indicator | | Factor 1 | Factor 2 | Factor 3 |
| --- | --- | --- | --- | --- |
| 1 | Boredom | **0.358*** | 0.145* | -0.026 |
| 2 | Addictive | **0.498*** | 0.028 | 0.215* |
| 3 | Entertainment | 0.009 | **0.516*** | 0.118 |
| 4 | Distraction | **0.521*** | -0.024* | 0.333* |
| 5 | Procrastination | **0.788*** | 0.180* | 0.001 |
| 6 | Money | -0.317* | 0.004 | **0.636*** |
| 7 | Socialise | -0.031 | **0.779*** | 0.009 |
| 8 | Communication | -0.010 | **0.889*** | -0.040 |
| 9 | Seeking help | 0.000 | 0.260* | **0.577*** |
| 10 | Observing others | 0.126* | **0.491*** | 0.212* |

*Note*. Asterisk indicates significant loading on factor; bold text indicates an item retained for the factor.

**Table S3**

*Geomin rotated loading for Exploratory Factor Analysis (two-factor) (N = 3410).*

| Indicator | | Factor 1 | Factor 2 |
| --- | --- | --- | --- |
| 1 | Boredom | **0.362*** | 0.074* |
| 2 | Addictive | **0.633*** | -0.008 |
| 3 | Entertainment | 0.083* | **0.535*** |
| 4 | Distraction | **0.700*** | -0.024 |
| 5 | Procrastination | **0.764*** | 0.069 |
| 6 | Money | 0.007 | 0.218* |
| 7 | Socialise | -0.010 | **0.781*** |
| 8 | Communication | -0.002 | **0.858*** |
| 9 | Seeking help | 0.295* | 0.386* |
| 10 | Observing others | 0.254* | **0.512*** |

*Note*. Asterisk indicates significant loading on factor; bold text indicates an item retained for the factor.

**Table S4**

*Geomin rotated loading for Exploratory Factor Analysis with boredom removed (N = 6820).*

| Indicator | | Factor 1 | Factor 2 |
| --- | --- | --- | --- |
| 1 | Addictive | **0.674*** | -0.033 |
| 2 | Entertainment | 0.080* | **0.505*** |
| 3 | Distraction | **0.694*** | -0.002 |
| 4 | Procrastination | **0.702*** | 0.130* |
| 5 | Money | 0.059 | 0.167* |
| 6 | Socialise | 0.003 | **0.780*** |
| 7 | Communication | -0.022 | **0.849*** |
| 8 | Seeking help | 0.330* | 0.317* |
| 9 | Observing others | 0.255* | **0.502*** |

*Note*. Asterisk indicates significant loading on factor; bold text indicates an item retained for the factor.

**Figure S1**

*Latent factor loadings - Confirmatory Factor Analysis of two-factor with boredom removed (N = 6820).*

Note. CFI = .978, TLI = .964, RMSEA = .046, SRMR = .039.

**Figure S2**

*Latent factor loadings - Confirmatory Factor Analysis of two-factor (N = 6820)*

**Figure S3**

*Latent factor loadings - Confirmatory Factor Analysis of two-factor (N = 3410)*

*Note*. CFI = .973, RMSEA = .043, SRMR = .041

**Table S5**

*Covariance between the Outcomes of the SEM Model with the Covariates (N = 5,540)*

|  | **Anxiety** | **Depression** | **Well-being** |
| --- | --- | --- | --- |
| **Depression** | .567** |  |  |
| **Well-being** | -.391** | -.497** |  |
| **Resilience** | -.430** | -.356** | .415** |

*Note.* ** p-value ≤ .001

**Figure S4**

*Illustration of the model fitted with gender and deprivation decomposed into two parts.*

*Note.* For clarity purposes, the fitted model was decomposed into 2 parts, and the covariances between variables were not drawn. A) illustrates the avoidant-escapist and social-recreational latent variables for social media and the paths between avoidant-escapist and social-recreational social media use on the outcomes of interest, B) illustrates the paths between gender and deprivation to avoidant-escapist and social-recreational social media use and the outcome of interest, and C) illustrates the complete fitted model. All of the paths drawn were significant with a *p*-value < .05

**Figure S5**

*Illustration of the model fitted (N = 3410).*

*Note*. CFI = .945, RMSEA = .057, SRMR = .054

**Table S6**

*Covariance between the Outcomes of the Model with the Covariates (N = 2,775)*

|  | **Anxiety** | **Depression** | **Well-being** |
| --- | --- | --- | --- |
| **Depression** | .546** |  |  |
| **Well-being** | -.379** | -.485** |  |
| **Resilience** | -.431** | -.336** | .410** |

*Note.* ** *p*-value ≤ .001

**Figure S6**

*Illustration of the model fitted with gender and deprivation decomposed into two parts (N = 2,775).*

*Note.* CFI = .951, RMSEA = .050, SRMR = .048. For clarity purposes, the fitted model was decomposed into 2 parts, and the covariances between variables were not drawn. A) illustrates the avoidant-escapist and social-recreational latent variables for social media and the paths between avoidant-escapist and social-recreational social media use on the outcomes of interest, B) illustrates the paths between gender and deprivation to avoidant-escapist and social-recreational social media use and the outcome of interest, and C) illustrates the complete fitted model. All of the paths drawn were significant with a *p*-value < .05.

**Table S7**

*Comparison of participants included in the covariate-adjusted model and those excluded due to missing covariate data.*

| Characteristic | Included N=5,540  Mean (SD) | Excluded N=1,280  Mean (SD) |
| --- | --- | --- |
| Age | 16.7 (0.69) | 16.9 (0.92) |
| Sex | 3503 / 63.2% (women)  2037 / 36.8% (men) | 547 / 42.7% (women)  329 / 25.7% (men)  404 / 31.6% (missing) |
| Anxiety | 6.72 (4.10) | 8.12 (4.37) |
| Depression | 5.90 (3.23) | 7.18 (3.55) |
| Wellbeing | 21.4 (3.33) | 20.1 (3.36) |
| Resilience | 3.07 (0.77) | 2.81 (0.80) |
